# Cell Signaling in Neurodegeneration

**DOI:** 10.3390/ijms22168978

**Published:** 2021-08-20

**Authors:** José L. Zugaza

**Affiliations:** 1 Achucarro Basque Center for Neuroscience, Science Park of the UPV/EHU, 48940 Leioa, Spain; joseluis.zugaza@ehu.es; Tel.: +34-946012256; 2 Department of Genetics, Physical Anthropology and Animal Physiology, University of Basque Country, UPV/EHU, 48940 Leioa, Spain; 3 Ikerbasque, Basque Foundation for Science, Plaza Euskadi 5, 48009 Bilbao, Spain

Neurodegenerative diseases are characterized by the progressive loss of specific subsets of neurons. The original trigger of neuronal death is still unknown, although aging is considered a risk factor for neurodegenerative diseases. Many of these disorders share an abnormal accumulation of misfolded peptides or proteins in the brain and spinal cord. These deposits of insoluble peptides or proteins could accumulate with time, and they could be more toxic when neurons are old. The accumulation of neurodegeneration-related peptides or proteins, such as Aβ_1–42_ peptide, hyperphosphorylated Tau protein or α-synuclein, among others, leads to the alteration of the neuronal and glial intracellular pathways. The consequences of this modified intracellular signaling can manifest at the level of protein quality control, dysfunctional mitochondrial homeostasis, autophagy and lysosomal dysregulation, abnormal presence of stress granules, synaptic toxicity, neuroinflammation or maladaptive innate immune response.

The Special Issue “*Cell Signaling and Neurodegeneration*” of the *International Journal of Molecular Sciences* covers eight original articles and six reviews that provide new insights regarding different signaling pathways that can be involved in neurodegenerative diseases.

Lenzi et al. investigate the role of clusterin (a molecular chaperone) in the -synuclein aggregation process. They report that clusterin is a key protein of the biochemical response conducted by the cell in order to regulate α-synuclein expression. They further suggest that clusterin is involved in the dynamic α-synuclein aggregation process [1].

Abdullah et al., studying the stimulator of interferon genes (STING) pathway in cell death induced by elevated stress, describe that H_2_O_2_-induced activation of the STING pathway is protective against cell death. They reveal that STING maintains efficient autophagy flux and protecting against H_2_O_2_-induced cell death. This investigation points out an important role for the STING pathway in the underlying cellular mechanisms contributing to the pathogenesis of neurological disorders [2].

Dash et al. create a gene profile of amyotropic lateral sclerosis (ALS) by analyzing the differentially expressed genes, the Kyoto Encyclopedia of Genes and Genomes pathways, the interactome and the transcription factor profiles that would identify altered molecular/functional signatures and their interactions at both transcriptional (mRNAs) and translational levels. The authors find that different genetic ALS forms are singular diseases rather than part of a common spectrum. This is crucial for individualized medicine approaches in ALS [3].

Avchalumov et al. examine whether acute methamphetamine treatment requires dopamine receptor type 1 activation in order to alter high-frecuency stimulation (HFS)-induced LTP in the dorsal striatum. They reveal that aberrant dopamine receptor type 1 function in the dorsal striatum by methamphetamine produces synaptic depression in the dorsal striatum. Therefore, the inhibition of dopamine receptor type 1 function may correlate with normalizing synaptic plasticity in the dorsal striatum and reducing the reinforcing properties of methamphetamine [4].

Ruiz et al. show that the transient integrated stress response induced by NMDA is followed by an ATF4-independent peIF2α dephosphorylation. However, Sephin1 strongly attenuates NMDA-induced excitotoxicity whereas it is ineffective against ER stress-produced neuronal death. The authors find that Sephin1 reduces NMDA-triggered cytosolic Ca^2+^ load and calpain activity and provides neuroprotection against excitotoxicity in an ISR-independent manner [5].

Tinelli et al. determine the level and function of circulating endothelial progenitor cells (cEPCs) in a cohort of Italian Moyamoya angiopathy patients in order to consider if cEPCs may be qualified as a potential pathogenic marker or just an epiphenomenon of Moyamoya angiopathy. This patholgy is a rare, chronic and disabling cerebrovascular disease with a prevalence of 0.086–10.5/100,000. They conclude that cEPCs level more than endothelial progenitor cell functionality seems to be a potential marker of Moyamoya angiopathy [6].

Bayer et al. investigate whether DNA methyltransferase 1 (DNMT1), which acts on neuronal survival in the aged brain, affects critical aspects of the proteostasis network. They found that DNMT1 negatively impacts retrograde trafficking and autophagy, with both being involved in the clearance of aggregation-prone proteins by the aggresome–autophagy pathway [7].

Scremin et al. focus their investigation on the role of ORAI2, a key store-operated Ca^2+^ entry (SOCE) component, in modulating SOCE in human neuroglioma cell line H4. The authors suggest that ORAI2 downregulation can become a potential tool to rescue defective SOCE in Alzheimer’s disease, while preventing plaque formation [8].

Martínez Cué and Rueda provide an overview of the signaling pathways implicated in each of the main neuropathological aspects of Alzheimer’s disease in individuals with and without Down syndrome as well as the interrelation of these pathways. In the fourth decade of life, all individuals with Down Syndrome develop neuropathology identical to that found in sporadic Alzheimer’s disease, including the development of amyloid plaques and neurofibrillary tangles, loss of neurons and synapses, reduced neurogenesis, enhanced oxidative stress, mitochondrial dysfunction and neuroinflammation. Down syndrome can be considered a useful model to study AD etiopathology and to search for new therapeutic strategies [9].

Arrazola et al. go deeper into the dysregulated/aberrant signaling pathways controlled by the small GTPaese of the Ras superfamily that culminate in neurodegeneration. The authors specifically focused on the two most studied families of the Ras superfamily: Ras and Rho. They summarize the latest findings of small GTPases of the Ras and Rho families in neurodegeneration in order to highlight these small proteins as potential therapeutic targets capable of slowing down different neurodegenerative diseases [10].

Grant and DeMorrow, in this review, provide a detailed discussion about the findings of recent studies highlighting bile acid-mediated therapies and bile acid-mediated signaling and the roles they play in neurodegenerative and neurological diseases [11].

Alcover-Sánchez et al. offer a thorough review of the tight relation between R-Ras 1 and R-Ras 2 GTPases and myelination processes and discuss its potential as novel elements of crosstalk between the pathways. They conclude that a better understanding of the crosstalk elements orchestrating myelination mechanisms is essential to identify new potential targets to mitigate neurodegeneration [12].

Sanz and García-Gimeno extensively review the recent progress made in the field of “Reactive glia inflammatory signaling pathways and epilepsy”, focusing on the main glial signaling pathways involved in neuroinflammation, how they are affected in epileptic conditions, and the therapeutic opportunities they offer to prevent these disorders [13].

Filipek and Leśniak provide a detailed discussion about “S100A6 and its brain ligands in neurodegenerative disorders”. The authors focus on the expression/localization of S100A6, CACYBP/SIP and SGT1 proteins in various brain structures and on their possible involvement in neurodegenerative diseases, such as Alzheimer’s disease, amyotrophic lateral sclerosis, epileptogenesis, Parkinson’s disease, Huntington’s disease, and others [14].
